# A systematic review of psychosocial functioning and quality of life in older people with bipolar disorder

**DOI:** 10.1016/j.jadr.2022.100371

**Published:** 2022-07

**Authors:** Dr Elizabeth Tyler, Professor Fiona Lobban, Mr Bogdan Hadarag, Professor Steven Jones

**Affiliations:** a Spectrum Centre for Mental Health Research, Division of Health Research, Lancaster University, Lancaster, UK; b Spectrum Centre for Mental Health Research, Division of Health Research, Lancaster University, Lancaster, UK; c Division of Health Research, Lancaster University, Lancaster, UK

**Keywords:** Bipolar disorder, Older adult, Psychosocial functioning, Quality of life, Systematic review

## Abstract

**Background:**

There is evidence to suggest that older people with Bipolar Disorder (BD) are more likely to demonstrate poor levels of functioning and score lower on well-being scales compared to non-clinical controls, even when in remission (Depp et al., 2006). To our knowledge, this is the first review paper to identify how quality of life and functioning has been measured in an older adult BD population.

**Methods:**

We conducted a systematic review of studies including a quantitative measure of psychosocial functioning or quality of life and older people over the age of 50 with a formal diagnosis of BD I or II.

**Results:**

Eleven studies (*N* = 726, mean age range 59.8 to 71.1) were included in the review, demonstrating a significant lack of research in the area compered to younger people with BD. The most commonly used measure of functioning was the Global Assessment of Functioning Scale (GAF) and results indicated that older adults with BD demonstrate a wide range of functioning.

**Limitations:**

The review used a comprehensive and systematic search strategy, however, very few eligible studies were available for review. The pooled analyses and reported means must be interpreted with caution due to the relatively small sample sizes.

**Conclusions:**

Older people with BD present with a wide range of functioning, ranging ‘major impairment’ to ‘superior’ scores. No existing validated measure assessing the psychosocial functioning or quality of life of older people with BD could be identified. Such a tool should be developed for use in future research.

## Highlights

• The Global Assessment of Functioning (GAF; Endicott et al., 1976) is the most widely used functioning measure used with older adults with bipolar disorder.
• Older adults with bipolar disorder demonstrate a wide range of functioning.
• No existing validated measure assessing the psychosocial functioning or quality of life of older people with BD could be identified.
• There is a significant lack of research in the area of older people with bipolar disorder compared to the younger population.

## Background and rationale

Bipolar Disorder (BD) is a mood disorder, characterised by episodes of depression and mania or hypomania, affecting approximately 2.4% of the global population (Merikangas et al., 2011). BD is classified as a lifelong, recurrent condition, associated with functional decline and a reduction in quality of life (Michalak et al., 2005; Bonnín et al., 2012). It has been ranked as one of the top 20 causes of the global disease burden (Global Burden of Disease Study., 2013). Previous studies have shown there is considerable functional impairment in adults with BD, even whilst their mood is stable (e.g., Rosa et al., 2008; Goetz et al., 2007; Strakowski et al., 2000). Similarly, studies have found that quality of life is impaired when individuals are both in episode and euthymic (e.g. IsHak et al., 2012 and Michalak et al., 2005).

Currently, the impact of living with BD in older adulthood has received far less attention than the adult population. There is evidence to suggest BD in later life may be more complex, with poorer cognitive functioning compared to non-clinical older adults (Gildengers et al., 2007), which may impact on functioning and lead to social, domestic, recreational and financial difficulties (Chen et al., 2017). There are also high levels of medical co-morbidity, (Lala et al., 2012, Tsai et al., 2009), with a higher risk for cardiovascular and respiratory conditions compared to aged matched controls (Rise et al., 2016). This may impact on quality of life as individuals are not able to engage in activities that they previously found enjoyable (Tyler et al., 2021). Depp et al. (2006) found that older people with BD were more likely to demonstrate poor levels of functioning and score lower on well-being scales compared to non-clinical controls, even when in remission. The negative effects on a person's functioning and quality of life can not only impact on the individual, but also the caregivers. The role can be extremely challenging and impact upon the caregiver's own quality of life (dos Santos et al., 2017).

The report from the International Society for Bipolar Disorders Task Force (Sajatovic et al., 2015) cites the lack of research and service development focused on older adults with BD. Given that the number of older people living with BD will increase dramatically over the next few decades due to our ageing population (United Nations, 2019) and that there is improved awareness of the condition (Hein et al., 2020), it is important to increase our understanding of BD in later life. Sajatovic et al. (2015) stipulate that our understanding must progress from generalising information from mixed age groups to studies developed specifically for the older adult BD population.

To address the lack of research in the area, the present study aimed to identify how psychosocial functioning and quality of life have been measured in older adults with BD. Once studies have been identified, data will be extracted to summarise levels of functioning and quality of life across studies that have included older people with BD. The purpose of this is to increase our understanding of the condition and help identify whether there are any measures that have been psychometrically examined for reliability or validity using an older adult BD sample for future research with this population.

### Aims and objectives

The current study aims to explore ways to assess quality of life and psychosocial functioning in older adults with BD. The objectives are to identify what measures have been used to assess quality of life and functioning with older people with BD and to describe the distribution of psychosocial functioning and quality of life scores for widely used measures.

### Research questions

1 What measures have been used to assess quality of life and psychosocial functioning in older adults with BD?
2 What is the distribution of psychosocial functioning and quality of life scores for older people with BD for the most widely used measures?

## Methods

### Protocol registration

The protocol was pre-registered on PROSPERO 2020 CRD42020200169. Available from:https://www.crd.york.ac.uk/PROSPERO/display_record.php?RecordID=200169

### Eligibility criteria

#### Inclusion

##### Studies were included if they

• Included a sample of individuals diagnosed with BD I or II with a formal diagnosis according to Diagnostic and Statistical Manual (DSM-III, DSM-IIIR, DSM-IV, DSM-IV-TR & DSM-V) or the International Classification of Diseases (ICD-9 or ICD-10) ***or*** a sample of mixed diagnoses which reported the scores of those with BD separately
• Included participants over the age of 50 ***or*** a sample of mixed ages which reported the scores of those over the age of 50 separately (based on the International Society for Bipolar Disorders Task Force (Sajatovic et al., 2015) recommendations to define older adults with BD ≥50 years).
• Included a quantitative measure of psychosocial functioning or quality of life

Were published in a peer-reviewed journal as a full article or short report.

### Exclusion criteria

#### Studies were excluded if they were

• Editorials, comments, letters to the editor, book chapters, case series, or dissertations/theses (i.e., grey literature).
• Not written in the English language due to no resources for translation.

### Search methods and information sources

The review adopted a comprehensive search strategy. Search terms were informed by previous Cochrane reviews for BD (e.g. Justo et al., 2007; Morriss et al., 2007) and functioning (Crotty et al., 2010) and systematic reviews investigating quality of life (e.g. Warkentin et al., 2014). Limiters in search engines were set to include peer reviewed studies, studies in the English Language, human participants only. There were no limiters set on publication date.

## Search terms

### Group one

“bi polar” OR “bi-polar” OR bipolar OR “mania*” OR “hypomani*” OR “mood disorder*” OR “mood disturbance*” OR “mood swing*” OR “affective disorder*” OR “affective illness*”

### Group two

“older adult” OR “older person” OR “old age” OR “elderly” OR “elderly person” OR “senior” OR “geriatric*” OR “retire*” OR “pension*” OR "over 50″ OR “older adult” OR “older person” OR “old age” OR “elderly” OR “elderly person” OR “senior” OR “geriatric*” OR “retire*” OR “pension*”

### Group three

“psycho social” OR “psycho-social” OR “psychosocial” OR “psychological functioning” OR “social functioning”

### Group four

“health-related quality of life” OR “health related quality of life” OR “quality of life measure” OR “QOL”

The group terms were also combined with the appropriate MeSH headings / subject headings in each database. The search method was as follows: group one AND group two AND group three OR group four.

Databases Medline, PSYCH-INFO, AMED and CINAHL were chosen following discussion with a University of Lancaster Librarian as their topic areas were within the scope of the review. Highly relevant papers were identified by the research team and test searches took place in February 2021 to ensure they were retrieved using the search strategy. The final database search took place at the end of April 2021. The reference lists of included articles were screened to identify any more potential eligible papers for the review.

### Study selection

The lead reviewer (ET) and a second reviewer (BH) independently screened all of the titles and abstracts. Any inconsistencies were shared with the wider team and discussed until an agreement was attained. Cohen's Kappa was used to assess the agreement between the two reviewers at the title and abstract screening stage.

The lead reviewer (ET) checked eligibility of all full texts using the inclusion and exclusion criteria. Additionally, the second reviewer (BH) independently assessed 30% of full texts. Extracted information included; type of study, study sample (e.g., age range, diagnosis, setting), the specific measure of psychological functioning or quality of life, the mean and/or median, standard deviation and/or range of scores. Please see supplementary information for an example of the data extraction form.

### Data analysis

The mean and/or median, standard deviation and/or range of scores from each included study sample were extracted from the included articles (see table 1). Where studies used the same measure, a cross study mean and SD was calculated to identify the distribution of scores across the studies. The means and SDs were pooled and weighted based on their study sample size according to Cohen's formula (Cohen, 1988; Zientek and Yetkiner, 2010).

**Table 1:** Summary of key study characteristics.

Authors and date	Study title	Study location	Study type	N	Age (s.d)	Gender% female	Diagnosis / Classification system	Current mood state	Quantitative measure	Mean (s.d)	Median (IQR)
Comes et al. (2017)	Functional Impairment in Older Adults With Bipolar Disorder	Barcelona, Spain	Observational, cross-sectional	33	68.7 (8.5)	51.5%	A formal diagnosis of BDI or BD II according to the DSM-IV	Euthymic	Functioning Assessment Short Test (FAST)	19.2 (11.4)	Not available from paper
Dautzenberg et al. (2016)	The care needs of older patients with bipolar disorder	Amsterdam, Netherlands	Cross-sectional	78	68.5(7.8)	48.7%	A formal diagnosis of BDI or BD II according to the DSM-IVTR	60% of the sample were in remission	The Global Assessment of functioning (GAF) Manchester Short Assessment of Quality of Life (MANSA)	GAF = 65.0 (11.2) MANSA = 61.9 (8.2) 5.2 per item	Not available from paper
Depp et al. (2007)	Medication adherence skills training for middle-aged and elderly adults with bipolar disorder: development and pilot study	San Diego, California	Quasi-experimental clinical trial	21	60.0 (6.1)	24%	A formal diagnosis of BDI or BD II according to the DSM-IV	Unknown	Short-Form of the Medical Outcomes Study Quality of Life Scale (SF-36)	SF-36 Physical functioning - 70.3 (24.4) SF-36 Role limitations – physical health - 59.4 (41.7)	Not available from paper
Dols et al. (2014)	Psychiatric and Medical Comorbidities: Results from a Bipolar Elderly Cohort Study	Amsterdam, Netherlands	Cross-Sectional	101	69.0 (7.8)	53.4%	A formal diagnosis of BDI or BD II according to the DSM-IV	Unknown	Global Assessment of Functioning (GAF)	63.1 (11.8)	Not available from paper
Liao et al. (2020)	Differences in outcomes between older community-dwelling patients with bipolar disorder and schizophrenia with illness onset at young age	Taipei, Taiwan	Cross-Sectional	113	59.8 (5.5)	59%	A formal diagnosis of BDI or BD II according to the DSM-IV	A score ≤5 on the Young Mania Rating Scale and Hamilton Depression Rating Scale total score <7 continuously for 2 months	Global Assessment of Functioning (GAF) The Strauss–Carpenter Scale (SCS).	GAF = 75.7 (10.2) SCS = 12.3 (3.0)	Not available from paper
Martino et al. (2018)	Neurocognitive heterogeneity in older adults with bipolar disorders	Buenos Aires, Argentina	Cross- Sectional	66	63.7 (8.0)	68.2%	A formal diagnosis of BDI or BD II according to the DSM-IV	Euthymic	The General Assessment of Functioning (GAF)	77.8 (10.8)	Not available from paper
Martino et al. (2013)	Neurocognitive functioning in early-onset (EO) and late-onset (LO) older patients with euthymic bipolar disorder	Buenos Aires, Argentina	Cross- Sectional	EO = 20 LO = 20	EO = 69.1 (6.7) LO = 66.9 (6.6)	EO = 90% LO = 75%	A formal diagnosis of BDI or BD II according to the DSM-IV	Euthymic	The General Assessment of Functioning (GAF)	EO = 76.4 (12.8) LO = 72.5 (9.9)	Not available from paper
Orhana et al. (M 2018)	The relationship between cognitive and social functioning in older patients with bipolar disorder	Amsterdam, the Netherlands	Cross- Sectional	63	66.0 (10.0),	49%	A formal diagnosis of BDI or BD II according to the DSM-IVTR	Unknown	Social and Occupational Functioning Assessment Scale (SOFAS) The Social Participation Scale (SPS)	Not available from paper	65 (15) 35–85 12 (4), 5–17
Parikh and Panse (2020)	Quality of life in elderly bipolar disorder patients	Maharashtra, India	Cross- Sectional	100	68.2 (5.8)	41%	A formal diagnosis of BD according to the ICD‑10 DCR criteria	Behaviourally stable during clinical interview	World Health Organization quality of life‑BREF	53.4	Not available from paper
Tsai et al. (2007)	Cognitive impairment in later life in patients with early-onset bipolar disorder	Taipei, Taiwan	Cross- Sectional	52	66.0 (6.5)	75%	A formal diagnosis of BDI according to the DSM-IV	Euthymic	Global Assessment of Functioning (GAF) Strauss–Carpenter score (SCS)	GAF = 69.6 (12.2) SCS = 10.8 (3.4)	Not available from paper
Tsai et al. (2009)	Cognitive Dysfunction and Medical Morbidity in Elderly Outpatients With Bipolar Disorder	Taipei, Taiwan	Case– control study	59	71.1 (5.9)	66.1%	A formal diagnosis of BDI according to the DSM-IV	Euthymic	Global Assessment of Functioning (GAF) scale Community Psychiatric Rating Scale (CPS)	GAF = 68.0 (10.8) CPS = 13.9 (4.7)	Not available from paper

## Results

2663 titles were initially identified (once duplicates were removed) and screened by the lead reviewer and an independent secondary reviewer (BH) by title and abstract. There was substantial agreement between the lead reviewer and secondary reviewer (κ = 0.631;95% CI, 0.564 to 0.699, p < .0005).

**Fig. 1:**
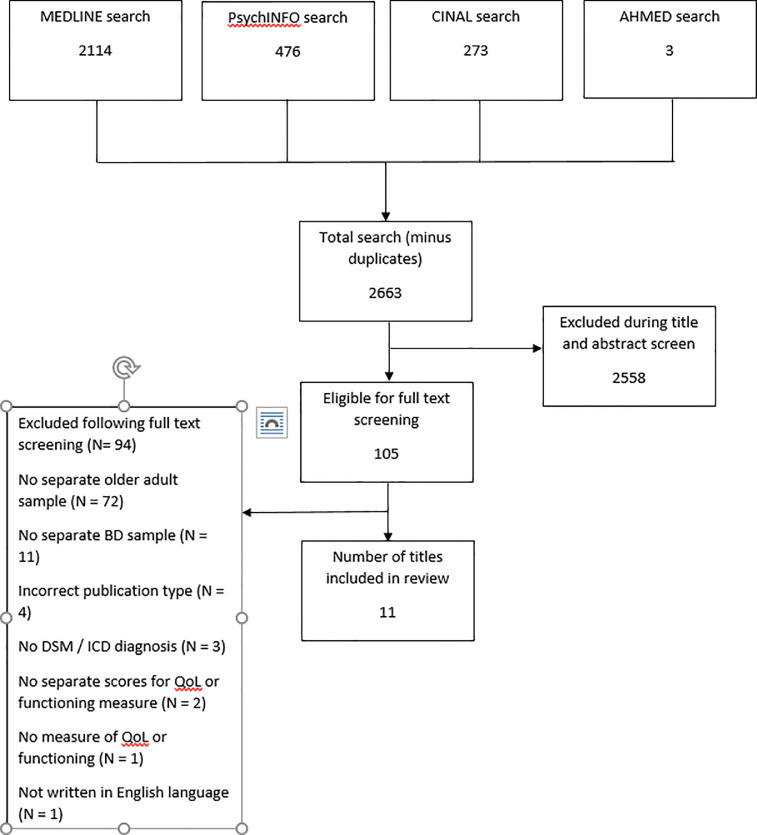
Flow diagram.

105 papers eligible for full text screening, 33% of these were screened by the secondary reviewer with 100% agreement. The final number of papers included in the review was 11. The most common reason for exclusion at this stage was not reporting results for the older people taking part in the study. The number of studies excluded are shown in Fig. 1.

### Description of included studies

11 papers were included in the review (see Table 1) with a total of 726 participants, including nine cross-sectional studies (Comes et al., M 2017; Dautzenberg et al., 2015; Dols et al., 2014; Liao et al., 2020; Martino et al., 2018, 2013;Orhan et al., 2018 Nov; 240, Parikh and Panse, 2020; Tsai et al., 2007), one case-control study (Tsai et al., 2009) and one quasi-experimental clinical trial (Depp et al., 2007). Studies were carried out in countries across the world including; the US, Taiwan, India, Argentina, the Netherlands and Spain. Sample sizes varied from 20 to 113.

Participants mean ages ranged from 59.8 (s.d. 5.5) to 71.1 (s.d. 5.9) years with seven out of 11 studies having higher proportions of women than men (range from 52% to 90%). Ten out of the 11 studies included individuals diagnosed with BD I or II according to Diagnostic and Statistical Manual (DSM-IIIR, DSM-IV, DSM-IV-TR) and one study used the International Classification of Diseases (ICD-10 DCR) criteria.

### Question 1

*What measures have been used to assess quality of life and psychosocial functioning in older adults with BD?*

11 papers reported using 10 different measures of psychosocial functioning and quality of life since 1997, which is the date that the first paper included in the review was published (see Table 2). This includes seven measures of functioning and three measures of quality of life. The most commonly used measure of functioning was the Global Assessment of Functioning Scale (GAF) which was used in seven of the papers. The second most used measure was the Strauss-Carpenter Scale (SCS) which was used in two of the studies.

### Question 2

*What is the distribution of psychosocial functioning and quality of life scores for older people with BD?*

The most frequently used measure was the Global Assessment of Functioning (GAF; Endicott et al., 1976) which is used to rate social, occupational, and psychological functioning. The GAF measures how much an individual's symptoms affect their day-to-day life on a scale of 0 to 100. The higher the score on the GAF, the better the person's level of functioning. The pooled GAF score (see Table 3) of 70.18 (s.*d* = 11.10), is based on data from 509 older people with BD and indicates an individual who has*; ‘some mild symptoms or some difficulty in social, occupational or school functioning, but generally functioning pretty well and has some meaningful interpersonal relationships*’ (American Psychiatric Association, 1994). The pooled analysis for the GAF indicates that 68% of the sample scored between 81.28 and 59.08 (one standard deviation above / below the mean), 13.5% scored between 81.28 and 92.38 and 47.98 and 59.08 (two standard deviations above / below the mean) and 2.5% scored between 92.38 and 103.48 and 47.98 and 36.88 (three standard deviations above / below the mean). However, there is a ceiling effect as individuals cannot score above 100 on the scale. A score of between 71 and 80 represents someone who has “*no more than a slight impairment in social, occupational or school functioning (*e.g.*, temporarily falling behind in school work) which* significantly contrast with individuals scoring lower on the scale with a score of between 31 and 40 representing ‘*some impairment in reality testing or communication, or major impairment in several areas such as work or school, family relations, judgement, thinking or mood”.*

**Table 2:** List of functioning and quality of life measures used in the 11 papers.

Measure name	Overall number of uses
Global Assessment of Functioning	7
The Strauss–Carpenter Scale	2
Social and Occupational Functioning Assessment Scale	1
Functioning Assessment Short Test	1
The Social Participation Scale	1
Community Psychiatric Rating Scale	1
Short-Form of the Medical Outcomes Study Quality of Life Scale SF-36	1
Manchester Short Assessment of Quality of Life	1
World Health Organization quality of life‑BREF quality of life assessment	1

The Strauss–Carpenter Scale (SCS; Strauss and Carpenter., 1972) was used in two studies (Liao et al., 2020 and Tsai et al., 2007). It is a prognostic scale, measuring four areas of functioning; hospitalisation, work, social activity and symptoms, scored out of a possible 16, with an established cut-off point of 14 for remission (Alberich et al., 2016). The pooled SCS score (see Table 3) from 165 older people with BD was 11.97, below the cut-off point for remission. The pooled analysis indicates that 68% of the sample scored between 15.09 and 8.85 (one standard deviation above / below the mean), 13.5% scored between 15.09 and 18.21 and 8.85 and 5.73 (two standard deviations above / below the mean) and 2.5% scored between 18.21 and 21.33 and 5.73 and 2.61 (three standard deviations above / below the mean). This finding suggests significant variability in scores on the SCS, with approximately 16% of individuals scoring above the cut-off point for remission according to Alberich et al. (2016).

**Table 3:** Pooled analysis of most widely used measures.

Measure	Number of studies with data	Combined N	Pooled age	Pooled mean	Pooled SD
The Global Assessment of Functioning	7	509	66.00	70.18	11.10
The Strauss-Carpenter Scale	2	165	61.74	11.97	3.12

Three quality of life measures were found across the 11 studies included. Scores on these measures ranged across the studies. Parikh and Panse (2020) used the WHOQOL‑BREF scale and the mean score was 53.40, indicating poor quality of life for older adults (Silva et al., 2014). The SF-36 was used in CA Depp et al. (2007) study and consists of eight scaled scores, transformed into a 0–100 scale, with the lower the score indicating more disability. The scores ranged from a low 31.3 (SF-36 Role limitations – emotional health) to a higher 70.3 (SF-36 Physical functioning). Quality of life was evaluated in Dautzenberg et al. (2015) study using the Manchester Short Assessment of Quality of Life (MANSA; Priebe et al. 1999) which rates satisfaction with various aspects of life. The MANSA score was 5.2 which is the mean of the 12 individual item scores, ranging from 1 (very dissatisfied) to 7 (very satisfied).

## Discussion

The aim of the review was to identify how quality of life and psychosocial functioning has been measured in older people with BD and to identify whether there were any measures that have been psychometrically examined for reliability or validity using an older adult BD sample for future research with this population. Once identified, we aimed to describe the distribution of scores for the most widely used measures to improve our understanding of how the condition presents in later life.

In this systematic review, we identified seven measures of psychosocial functioning and three measures of quality of life, across 11 research studies. This low volume of research contrast with findings from adults of working age with BD. Akers et al. (2019) identified 379 research studies reporting 38 different measures of social and occupational functioning, published since 1981. With regards to quality of life, a recent systematic review and meta-analysis of cross-sectional case-controlled studies including adults with BD found 23 eligible studies and reported four different measures of quality of life (Pascual-Sánchez et al., 2019).

The most frequently used measure in this review was the GAF, used in seven of the studies included. The GAF is a global measure of functioning not specifically validated for use with individuals with BD. Only two of the seven functioning measures identified, the SCS and the Functioning Assessment Short Test (FAST; Rosa et al., 2007) have been psychometrically examined for reliability or validity using a BD only sample. None of the three quality of life measures (WHOQOL‑BREF, SF-36 and MANSA) identified have been validated using a BD sample. Furthermore, none of the ten measures identified in this review have been psychometrically examined for reliability or validity in an older adult BD only sample.

The GAF was also the most frequently used measure found in Akers et al. (2019) review, with 166 overall uses since 1981 with working age adults. In the current review, the pooled average GAF score was 70.2 (s.*d* = 11.10), indicating individuals “*experiencing some mild symptoms or some difficulty in social, occupational or school functioning”.* The pooled score for working aged adults was 63.63 (s.*d* = 12.68) which falls into the same category as that for the older adults, albeit slightly lower. Similarly, to Akers et al. (2019), there was significantly variability across the scale, indicating that some older adults were presenting with *‘major impairments in several areas’*, however, others were scoring at the top of the scale with ‘*superior functioning in a wide range of activities”*’. These findings support the existing literature focused on working aged adults where BD is associated with significant impairments in areas of functioning such as work, family and social life (Sanchez-Moreno et al., 2009). There are, however, also a proportion of individuals with BD that maintain at least adequate levels of occupational and social functioning whilst living with the condition (MacQueen et al., 2001). There is also evidence to suggest that some individuals with BD exhibit higher levels of functioning compared to the general population and excel in areas such as creativity (Goodwin and Jamison, 2007; Johnson et al., 2012).

One advantages of the GAF is its wide-ranging scale, which allows representation for individuals scoring on the higher end of the scale. A recent study (Lomastro et al., 2020) with adults with BD, aged 18–65 years found that having a diagnosis of BD II, a higher educational level, and better performance in verbal memory, attention, and executive functions independently predicted high psychosocial functioning (scoring 90 or more on the GAF). Developing an understanding of the factors that influence higher functioning for older people with BD is important. This will increase our understanding of the condition in later life and help shape effective therapeutic interventions for those presenting with lower levels of functioning.

The GAF was eliminated from the DSM-5 in 2013 for a number of reasons, including the observations that the overall score often correlates with the person's severity of symptoms, rather than the levels of impairment (Gold, 2014). This has been supported by a number of studies that have found that the GAF might be mediated by symptoms (Samara et al., 2014; Suzuki et al., 2015). Interestingly, studies included in the present review where the sample is euthymic (indicating a neutral mood with few symptoms), have a higher average score on the GAF, representing a better level of functioning, compared to those where the current mood state was unknown. The Social and Occupational Functioning Assessment Scale (SOFAS; Rybarczyk, 2011) was developed from the GAF and included in the Diagnostic and Statistical Manual for Mental Disorders 4th Edition (American Psychiatric Association, 2000). The SOFAS gives a more accurate portrayal of functioning as it is independent of the person's severity of psychological symptoms (Rybarczyk, 2011). Only one of the studies (Orhan et al., 2018) included in the current review used the measure, however Aker's et al. (2019) found the SOFAS was used in 29 different research studies with working aged adults.

The SCS was used in two of the studies in the present review. In Aker's et al's 2019 review, they found the SCS had been used 18 times since 1981, with four uses in the past 10 years, demonstrating a reduction in its use over time. The SCS has now been psychometrically examined for reliability or validity using a BD only sample (Alberich et al., 2016) and therefore we may see an increase in the use of the measure in BD research. Alberich et al. (2016) established a cut-off score of 14 for remission, with a higher score indication better functioning. The pooled score for this review was 11.97, indicating individuals were still having some mild difficulties with functioning, and consistent with the pooled scored from the GAF. A disadvantage of the SCS is it does not provide a range of functioning levels only a cut off for remission. Therefore, scales such as the GAF and SOFAS have an advantage as they have a wide range of possible scores which can indicate both problematic and superior functioning, allowing more insight into the person's presenting difficulties.

Only three quality of life measures were found across the 11 studies included. The scores on the measures ranged across the studies. Parikh and Panse (2020) used the WHOQOL‑BREF scale and reported scores indicating poor quality of life for older adults, whereas Dautzenberg et al. (2015) used the MANSA which indicated moderate satisfaction with various aspects of life. Depp et al. (2007) used the SF-36 which consists of eight scales and reported a range of scores from low to higher levels of perceived health and well-being (Depp et al., 2007). Limited conclusions can be drawn from the findings due to the small number of studies including a quality of life measure and the inconsistent pattern of scores.

The findings from the current systematic review confirm previous reports that the impact of BD on older adults and its relationship with psychosocial functioning (Nivoli et al., 2014) and quality of life (Parikh and Panse, 2020) has received little attention. This finding for older adults with BD mirrors a review focused on the quality of life of older people in general staying at home as they age, rather than ageing in residential care. They found a small number of studies reported the assessment of quality of life (Vanleerberghe et al., 2017), even though a number of international action plans focused on ageing highlight the importance of improving quality of life for people as they age (World Health Organisation, 2015; Tesch-Roemer, 2012; Malva and Bousquet, 2016). A recent review found 44 available quality of life instruments for use with people with mental health problems (van Krugten et al., 2021). Only one of the 44 was developed specifically for older people, the World Health Organization Quality of Life Questionnaire – Older Adults Module (Power et al., 2005) and this was not used in any of the studies in this review. Therefore, it appears that there are a wide range of quality-of-life instruments available for younger people, compared to measures developed specifically for an older adult population.

### Recommendations for future research

The findings from the review indicate that there is significant variability on the both the quality of life and functioning measures. Future research should focus on understanding why some individuals score higher on quality of life and functioning measures. This which may provide some insight into the factors that influence superior functioning and quality of life. These findings may help shape effective therapeutic interventions for individuals scoring lower on the scales.

The systematic review did not find any evidence for an existing validated measure of psychosocial functioning or quality of life for older people with BD. There are existing measures that have been developed for an adult population such as the Bipolar Recovery Questionnaire (Jones et al., 2013) and the Quality of Life in Bipolar Disorder (QoL.BD) scale (Michalak and Murray, 2010). As highlighted above, international action plans (e.g., World Health Organisation, 2015) highlight the importance of improving quality of life as people age and therefore there needs to be an appropriate tool to measure this. It has also been suggested that for individuals with BD, functional outcomes are more meaningful measures of response to treatment compared to psychiatric rating scales (Keck, 2004). Therefore, a measure specifically for older people with BD must be developed for use with this population.

### Strengths and limitations

There are a number of strengths to this review. A comprehensive and systematic search strategy to identify relevant papers was used. All identified titles and abstracts were double screened by two reviewers, with substantial agreement. Thirty-three percent of eligible papers for full text screening were also screened by both reviewers, with 100% agreement. However, very few eligible studies were identified to review, therefore the pooled analyses and reported means must be interpreted with caution due to the relatively small sample sizes. Secondly, the mood state of included participants was unknown in a number of the studies. Participants may have been symptomatic which may have affected their score on the GAF and the pooled analysis for the study. Additionally, studies written in another language were excluded from the review due to resource constraints for interpretation and therefore the results may not be generalisable.

## Conclusion

The present systematic review identified very few studies reporting the assessment of psychosocial functioning and quality of life in older people with BD, especially when compared to working aged adults with BD (Akers et al., 2019). The GAF was the most frequently used measure of functioning, used in seven of the 11 studies included in the review. There was significantly variability across the scale, indicating that older people with BD are presenting with a wide range of functioning, consistent with working aged adults (Akers et al., 2019).

## Declarations

Competing interests: None declared.

## Funding

This review was funded by the National Institute for Health Research through the Doctoral Research Fellowship programme. Grant No DRF-2014–07–094.

## Author contributions

ET designed the study, wrote the protocol, conducted the literature searches, screened the articles, extracted the data, conducted the analysis and wrote the manuscript. SJ and FH contributed towards the study development and protocol, proofed, edited and approved the final manuscript. BH conducted the article screening, proofed and approved the final manuscript.

## Conflict of interest

The authors declared no conflicts of interest.
